# Airflow limitation as a risk factor for low bone mineral density and hip fracture: The Hordaland Health Study (HUSK) – 10 years follow-up of a general population

**DOI:** 10.3402/ecrj.v3.32214

**Published:** 2016-10-11

**Authors:** Trine Herland, Ellen M Apalset, Geir Egil Eide, Grethe S Tell, Sverre Lehmann

**Affiliations:** 1 Department of Clinical Science, University of Bergen, Bergen, Norway; 2 Department of Rheumatology, Haukeland University Hospital, Bergen, Norway; 3 Department of Global Public Health and Primary Care, University of Bergen, Bergen, Norway; 4 Centre for Clinical Research, Haukeland University Hospital, Bergen, Norway; 5 Department of Thoracic Medicine, Haukeland University Hospital, Bergen, Norway

**Keywords:** lung function, bone mineral density, hip fracture, airflow limitation, population study

## Abstract

**Aim:**

To investigate whether airflow limitation is associated with bone mineral density (BMD) and risk of hip fractures.

**Methods:**

A community sample of 5,100 subjects 47–48 and 71–73 years old and living in Bergen was invited. Participants filled in questionnaires and performed a post-bronchodilator spirometry measuring forced expiratory volume in 1 second (FEV_1_) and forced vital capacity (FVC). All attendants were invited to have a BMD measurement of the hip. During 10 years of follow-up, information on death was collected from the Norwegian Cause of Death Registry, and incident hip fractures were registered from regional hospital records of discharge diagnoses and surgical procedure codes.

**Results:**

The attendance rate was 69% (*n*=3,506). The prevalence of chronic obstructive pulmonary disease (COPD) (FEV_1_/FVC<0.7) was 9%. In multiple logistic regression, the lowest quartile of BMD versus the three upper was significantly predicted by FEV_1_/FVC<0.7 and FEV_1_% predicted (odds ratio [OR]: 1.58, 95% confidence interval [CI]: 1.11 to 2.25, and OR per increase of 10%: 0.92, 95% CI: 0.86 to 0.99, respectively). Hip fracture occurred in 126 (4%) participants. In a Cox regression analysis, FEV_1_% predicted was associated with a lowered risk of hip fracture (hazard ratio per increase of 10%: 0.89, 95% CI: 0.79 to 0.997).

**Conclusion:**

Airflow limitation is positively associated with low BMD and risk of hip fracture in middle-aged and elderly.

Hip fracture, defined as a fracture of the proximal femur, is a common event in the elderly, resulting in reduced quality of life and increased mortality (1). The number of new cases worldwide has been estimated at more than 1.6 million per year in subjects above 50 years of age (2). The annual incidence is highest in the Scandinavian countries and is estimated as 400 hip fractures per 1 million inhabitants (3). Osteoporosis is by far the most important risk factor for hip fractures; it commonly is characterised by reduced bone mass and disruption of bone architecture, resulting in increased bone fragility (4). Furthermore, osteoporosis is considerably more prevalent in persons with chronic obstructive pulmonary disease (COPD) than in healthy subjects, ranging from 9 to 69% in different cohorts (5), suggesting a higher risk of hip fractures in subjects with non-reversible airflow limitation.

Large national registry data indicate that hip fracture is a life-threatening event in patients with COPD, with a 50 to 70% higher risk of death than for subjects suffering these fractures without concomitant COPD (6, 7). The most common explanations for the association among COPD, osteoporosis, and fractures are corticosteroid medication, smoking, low body mass index (BMI), and physical inactivity (8). However, COPD is characterised by a sustained airflow limitation associated with enhanced systemic inflammation (9), which may contribute to increased bone degradation and osteoporosis (10, 11). Only a few studies have investigated whether there is an association between lung function and bone fractures per se, that is, independent of other known fracture risk factors. In a prospective population-based study in England, low forced expiratory volume in 1 second (FEV_1_) was associated with risk of hip fracture (12), and a cross-sectional Norwegian study demonstrated higher risk of vertebral deformities in COPD patients compared to a control group (13). Finally, in a large cross-sectional study of 3,030 COPD patients in Italy, the risk of fractures was related to the severity of the disease (14). These studies had, however, some limitations in study design, such as lack of post-bronchodilator spirometry values and information on physical activity (12), considerable selection bias of the clinical sample (13), and absence of a control group in the latter Italian study (14). Also, two of the studies (13, 14) reported vertebral fractures only, not hip fractures.

Our aim was to investigate whether non-reversible airflow limitation, measured by post-bronchodilator spirometry, is independently associated with reduced bone mineral density (BMD) and risk of hip fractures in community-dwelling, middle-aged and older adults.

## Materials and methods

### Study population

**Fig. 1:**
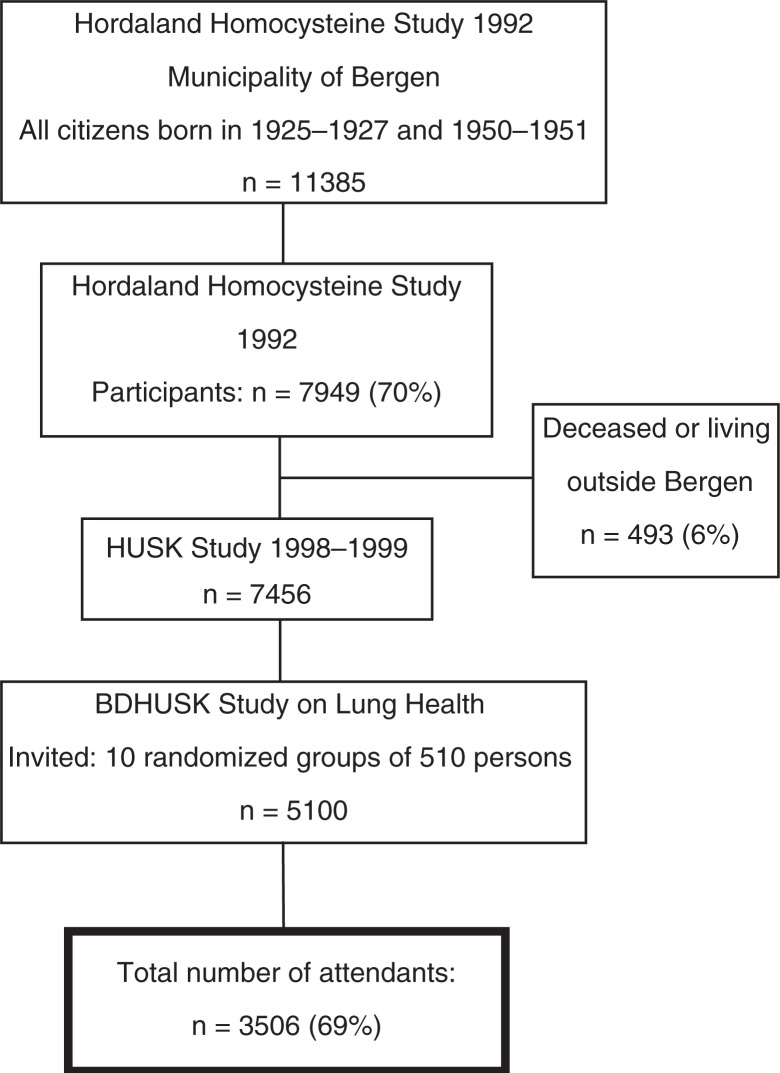
Flow chart of the participants of the Hordaland Health Study (HUSK) in Norway 1992–1999. BDHUSK = The BronchoDilator spirometry sub-study on Lung Health from the main Hordaland Health Study (HUSK).

The target population was all women and men born in 1925–1927 and 1950–1951 living in Bergen, Norway, as of 31 December 1992. A total of 70% (*n*=7,949) of these subjects participated in the initial cardiovascular study in 1993 (i.e. the Hordaland Homocysteine Study) (15). In 1998–1999, a follow-up study was conducted, that is, the Hordaland Health Study (HUSK). HUSK was a collaborative study between the Norwegian National Health Screening Service and the University of Bergen. A total of 7,456 participants still alive and living in Bergen were invited to participate. Due to resource limitations, random samples of 510 persons from each of the ten age–gender strata were drawn; a total of 5,100 subjects were invited to participate in the bronchodilation study (BDHUSK; Fig. 1). Participants in the BDHUSK received a postal questionnaire on general health issues, an invitation to meet at a survey centre for spirometry with bronchodilator testing – including a lung specific questionnaire, and, lastly, an invitation to undergo BMD measurement.

The study, including ascertainment of subsequent fractures, was approved by the Norwegian Regional Committee for Medical and Health Research Ethics, Health Region West.

### Outcome variables

A stationary, dual X-ray densitometer (EXPERT-XL; LUNAR Corp., Madison, WI, software versions 1.72 and 1.90) was used for BMD measurements. The left hip was scanned (except in those participants with a left hip prosthesis or former hip fracture). The results for BMD in total hip were reported (16). All incident hip fractures were registered from computerised records of discharge diagnoses from all hospitalizations in the region in the period between enrolment and 31 December 2009. Only hip fractures confirmed by a concurrent code of an adequate surgical procedure were included, as previously described (17). Time of death information was obtained from the Norwegian Cause of Death Registry (18). For the purpose of analysis, BMD was categorised in quartiles and dichotomised in the lowest quartile (low BMD) versus the three upper.

### Explanatory variables

Bronchodilator tests were guided by one trained technician using a dry wedge Vitalograph S (Vitalograph, Buckingham, UK) spirometer before, and 15 min after, inhalation of 400 µg of salbutamol, according to the Global Initiative on Obstructive Lung Disease (GOLD) guideline (19). Calibration of the equipment, including daily biologic control, was performed as described in a previous paper (20). The largest FEV_1_ and forced vital capacity (FVC) values from three acceptable spirograms, of which the two largest values were within 200 ml of each other, (21) were selected. The values of FEV_1_ and FVC were corrected to body temperature and pressure-saturated conditions (BTPS), and FEV_1_ was expressed as the percentage of predicted values using normative values from a Norwegian population (22). Post-bronchodilator airflow limitation was defined in accordance with the GOLD guideline (23) as either spirometric COPD (FEV_1_/FVC<0.7) with sub-classification into mild, moderate, or severe disease by level of the predicted FEV_1_ or as the newer clinical classification into severity grade A to D based on lung function, dyspnoea scale, and exacerbation history (24). Dyspnoea scale and exacerbation history were based on questionnaires filled out by the participants.

### Possible confounding variables

Information about smoking history, regular leisure time exercise, and use of inhaled or oral corticosteroid medication during the previous 12 months was obtained. Participants were categorised into never, current and ex-smokers. Pack years were calculated as: (mean number of cigarettes consumed daily/20)×years of daily smoking. Leisure time exercise was classified as light (no sweat/no shortness of breath) or hard (sweat/shortness of breath), and both were further categorised into: at least 1 h per week or less than 1 h per week. Weight and height were measured with light clothing and without shoes. BMI was categorised according to the World Health Organization standards (25) and used as a continuous variable in the multivariate analyses. Assessments of cognitive function were conducted in the elderly cohort using the Mini Mental Status Examination (MMSE) (20). An MMSE score of 10 to 12 was considered normal, whereas scores below 10 were reflected as cognitive dysfunction.

### Statistical analysis

Descriptive statistics were reported as percentages for categorical variables, the mean and standard deviation (SD) for continuous variables, and Kaplan-Meier estimates for time from inclusion to fracture. Subjects dying before end-of-follow-up without any fracture and subjects alive at end-of-follow-up were considered as censored observations. Groups were compared using the chi-square test or Fisher's exact test for categorical variables, Student's t-test for continuous variables, and the log-rank test for time to fracture. Dichotomised BMD, defined as the lowest quartile in each of four age and gender groups, was used as the response variable in multiple logistic regression and time to hip fracture in multiple Cox regression. We checked the assumption of proportional hazards using log-minus-log-survival plots. The results were reported using the odds ratio (OR) and the hazard ratio (HR), respectively, with a 95% corresponding confidence interval (CI). All multiple regression analyses were adjusted for age cohort, gender, BMI, smoking status (never, current, and ex-smokers), and use of corticosteroids (no, oral, and inhaled). Other explanatory variables significantly associated with an increased risk of hip fractures from the univariate analyses were included in the regression models with separate analyses for each lung function variable. Two-sided *p-values* less or equal to 0.05 were considered statistically significant. Statistical analyses were performed using SPSS (IBM SPSS Statistics 21).

## Results

The total number of respondents was 3,506. The participation rate was higher in the middle-aged (76%) than in the elderly (64%) cohort (*p*=0.001). There was no observed difference in participation between the genders (*p*=0.10).

A total of 3,305 subjects (94%) performed a satisfactory reversibility test and were included (characteristics given in Table 1), whereas 201 subjects (6%) failed the quality criteria and were excluded (26). Altogether 303 (9.2%) subjects were classified as having COPD at baseline, and a total of 126 (3.8%) experienced a hip fracture during follow-up. Throughout the observation period, 426 (12.9%) persons died. Among these, 33 subjects (7.7%) had suffered a hip fracture.

**Table 1:** Main characteristics of participants with and without COPD^a^ stratified by gender in the Hordaland Health Study, Norway, 1998–1999 (*n*=3,305)

	Women			Men		
		
	COPD^a^	Non-COPD		COPD^a^	Non-COPD	
Characteristics	*n* (%)	*n* (%)	*P*	*n* (%)	*n* (%)	*P*
Age cohort			<0.001			<0.001
47–48 years	16 (18.0)	783 (49.5)		24 (11.2)	682 (48.0)	
71–73 years	73 (82.0)	798 (50.5)		190 (88.8)	739 (52.0)	
Hip fracture			0.001			0.012
Yes	10 (11.2)	63 (4.0)		13 (6.1)	40 (2.8)	
Glucocorticoids						
Inhaled	17 (19.1)	58 (3.7)	<0.001	39 (18.2)	39 (2.7)	<0.001
Oral	5 (5.6)	9 (0.6)	<0.001	3 (1.4)	3 (0.2)	0.029^b^
Smoking						
*Status*			<0.001			<0.001
Never	18 (20.2)	758 (48.0)		14 (6.5)	418 (29.4)	
Former	30 (33.7)	419 (26.5)		114 (53.3)	685 (48.2)	
Current	39 (43.8)	367 (23.2)		85 (40.0)	297 (21.0)	
*Pack years*			<0.001			<0.001
0	18 (20.2)	758 (48.0)		14 (6.5)	418 (29.4)	
<10	17 (19.1)	358 (22.6)		21 (9.8)	357 (25.1)	
10–20	21 (23.6)	191 (12.1)		57 (26.6)	242 (17.0)	
20+	28 (31.5)	169 (10.7)		108 (50.5)	287 (20.2)	
BMI (kg/m^2^)			0.083			<0.001
<18.5	4 (4.5)	25 (1.6)		2 (0.9)	4 (0.3)	
18.5–24.9	50 (56.2)	770 (48.7)		114 (53.3)	519 (36.5)	
25.0–29.9	25 (28.1)	550 (34.8)		77 (36.0)	731 (51.4)	
30+	9 (10.1)	214 (13.5)		17 (8.0)	140 (9.9)	
Exercise						
No	18 (22.0)	183 (12.5)	0.003	31 (16.1)	155 (11.5)	0.015
Light	49 (59.8)	783 (53.7)		101 (52.3)	626 (46.6)	
Hard	15 (18.3)	493 (33.8)		61 (31.6)	563 (41.9)	

COPD: chronic obstructive pulmonary disease; BMI: body mass index; FEV_1_: forced expiratory volume in 1 s; FVC: forced vital capacity.

a COPD defined as post-bronchodilatory FEV_1_/FVC<0.7.

b Fisher's exact test if<5 expected subjects in one or more of the groups.

Among the subjects with approved lung function tests, 2,584 (78.2%) subjects consented to the BMD examination, constituting 1,315 elderly and 1,269 middle-aged subjects (within age group attendance 73.1% vs. 84.3%, respectively, *p*=0.001), and similarly the attendance by gender was 1,239 men and 1,345 women (75.8% vs. 80.5%, respectively, *p*=0.001). Smoking habits were not significantly different in the BMD group compared to the total study group.

### Relationship between airflow limitation and low BMD

The mean (SD) total hip BMD was lower in elderly participants compared to those that were middle-aged (Table 2) and lower in women compared to men (0.90 [0.15] g/cm^2^ vs. 0.99 [0.15] g/cm^2^, *p*<0.001. Further, BMD was lower in subjects with versus without COPD (0.88 [0.16] g/cm^2^ vs. 0.95 [0.16] g/cm^2^, *p*<0.001). In the multivariate analyses (Table 3), both indices of airflow limitation were associated with low BMD.

**Table 2:** Bone mineral density (BMD) at baseline in 1998–1999 (*n*=2,584)^a^ and hip fracture rates (*n*=3,305)^b^ during 10 years’ follow-up by age cohort in the Hordaland Health Study in 1998–1999

	Middle-aged (47–48 years)	Elderly (71–73 years)	*P*
BMD g/cm^2^, mean (SD)	1.00 (0.13)	0.89 (0.16)	0.001^c^
Hip fracture rate %	0.6	6.5	0.001^d^

BMD: bone mineral density; SD: standard deviation.

a 1,269 middle-aged and 1,325 elderly.

b 1,505 middle-aged and 1800 elderly.

c Student's t-test.

d Chi-square test.

**Table 3:** Post-bronchodilator lung function variables predicting low bone mineral density (BMD) of the hip^a^ in multiple logistic regression analysis. The Hordaland Health Study in 1998–1999 (*n=*2,584).

	Multiple logistic regression^c^		
	
Predictors^b^	OR	95% CI	*P*
FEV_1_/FVC			
≥0.7	1.00		0.011
<0.7	1.58	(1.11, 2.25)	
FEV_1_% predicted			
Per 10 increase	0.92	(0.86, 0.99)	0.017

FEV_1_: forced expiratory volume in 1 second; FVC: forced vital capacity; OR: odds ratio; CI: confidence interval.

a Defined as the lowest quartile in each of four age/gender groups among middle-aged (47–48 years) and elderly (71–73 years) women and men.

b The analyses are performed for each lung function variable separately and were not adjusted for the other index of airflow limitation.

c Adjusted for age, sex, smoking habits (never, ex-, and current smokers), BMI (continuous variable), glucocorticoids (no, inhaled, and oral) and exercise (no, light, and hard).

### Predictors of incident hip fractures during 10-years’ follow-up

In the multivariate analyses (Table 4), a 10% increase in post-bronchodilator FEV_1_ (expressed as% of predicted value) was significantly associated with an 11% reduction in hip fracture risk. When baseline BMD was added to the statistical models, low levels of BMD were strongly associated with incident hip fractures during follow-up, and the independent relationships between the lung function variables and fractures were no longer present (results not shown). In middle-aged subjects, FEV_1_/FVC<0.7 resulted in a 17 times higher HR for hip fracture during 10 years’ follow-up compared to middle-aged subjects with FEV_1_/FVC ≥0.7. In elderly subjects, FEV1/FVC<0.7 gave a 1.13 times higher HR for hip fracture compared to elderly with FEV_1_/FVC ≥0.7. In middle-aged subjects, each increase of 10 in FEV_1_% predicted resulted in a 53% lower HR for hip fracture during 10 years. In elderly subjects, each increase of 10 in FEV_1_% predicted gave an 8% lower HR for hip fracture.

**Table 4:** Results from multiple Cox regression analysis of hazard of hip fracture^a^ in 3,305 subjects in the Hordaland Health Study, 1998–1999, with 10 years’ follow-up

Explanatory baseline		Model without interaction^c^			Model with interaction^c^		
			
Spirometric variable^b^	Age cohort	HR	95% CI	*P*	HR	95% CI	*P* d
FEV1/FVC (<0.7 vs. ≥0.7)	47–48 years	1.35	(0.78, 2.32)	0.285	16.96	(3.94, 73.01)	0.001
	71–73 years				1.13	(0.64, 2.01)	
FEV1% predicted (continuous) per 10%	47–48 years	0.89	(0.79, 0.997)	0.045	0.47	(0.30, 0.72)	0.003
	71–73 years				0.92	(0.81, 1.03)	

FEV_1_: forced expiratory volume in 1 second; FVC: forced vital capacity; HR: hazard ratio; CI: confidence interval.

a Defined as the first fracture of the proximal femur that occurred during the observation period and confirmed by concurrent surgical procedure codes.

b The analyses were performed for each lung function variable separately and were not adjusted for the other index of airflow limitation.

c Adjusted for age cohort, sex, smoking habits (never, ex-, current smoker), body mass index (continuous variable), glucocorticoids (no, inhaled, and/or oral), and exercise (no, light, and hard).

d For test of interaction between age cohort and spirometric variable.

## Discussion

In this sample of community-dwelling, middle-aged and elderly men and women, non-reversible airflow limitation measured by spirometry was associated with low levels of BMD and with subsequent risk of hip fractures during the following 10 years. As expected, the baseline BMD of the hip was more strongly associated with risk of hip fracture than the airflow limitation.

### 

BDHUSK was a sub-study of the HUSK based on a large sample of the general population (27, 28). One well-trained technician guided the participants, securing high-quality spirograms (20). Information on risk factors for osteoporotic fractures was collected in the study (29), with highly valid methods for collection of the outcome variables using Dual-energy X-ray apsorptiometry (DXA) scanning and high-quality computerised records of discharge diagnosis confirmed by concurrent surgical procedure codes.

Blood samples of vitamin D were, however, not available. Vitamin D deficiency is related to osteoporosis and is highly prevalent in patients with COPD. In the Bergen COPD cohort study performed in the same geographic area as the current study, the prevalence of vitamin D deficiency was higher among COPD patients than in control subjects after adjusting for season, comorbidities, age, smoking, and BMI (30). Thus, a low level of vitamin D is a potential confounder not adjusted for in our study.

The current study adds to an increasing number of publications suggesting that smoking, low BMI, use of glucocorticoids, lack of exercise, and chronic airflow limitation are independent risk factors of osteoporosis and osteoporotic fractures (5, 31). In a British prospective study of general practice records of 14,800 subjects aged 42–81 years (12), low FEV_1_ was associated with fractures of the hip during a mean follow-up time of 7.7 years. In a Norwegian cross-sectional study (13), the prevalence of vertebral deformities in 465 COPD patients was almost twice as high as in the 462 controls and was related to disease severity in women after adjustment for other risk factors, suggesting that the lung disease itself had a specific effect. However, some important explanatory variables were lacking in the former population-based study, such as post-bronchodilator spirometry values, including the FEV_1_/FVC ratio, and data on physical activity. In the latter cross-sectional study, more than 50% of the screened COPD patients were excluded, indicating a possible selection bias. Also, the design was cross-sectional, and hip fracture was not used as an endpoint. Thus, our findings confirm and strengthen those from previous studies by using the major clinically relevant explanatory variables, including post-beta2 agonist measurements of airflow limitation.

Nevertheless, selection bias may have had some influence on our results. The participation rate and the proportion of acceptable reversibility tests in the elderly cohort were lower than in the middle-aged cohort. Furthermore, subjects with reduced levels of lung function, low body weight, and low physical activity were probably underrepresented, resulting in loss of an unknown number of high-risk subjects at baseline. Even though a previous population study from Hordaland County, including Bergen, showed that the relationship between smoking and lung disease remained unchanged with increasing response rate (32), a non-response bias might have played a role in the current study. This most likely resulted in an underestimation of the risk effects of several explanatory variables. Smoking is a known risk factor for hip fracture and both ‘ever smoking’ and pack years were associated with low BMD at baseline (results not shown). Surprisingly, we did not find a significant relationship between ‘ever smoking’ and later hip fracture. This might be explained by an excess mortality caused by smoking-related diseases during follow-up in these age cohorts because 389 (77.5%) of the 502 subjects who died were current or ex-smokers, but in the total study group at baseline, 61.6% were recorded as ever smokers.

Previous studies have indicated a relationship between cognitive dysfunction and increased risk of falling and thereby higher risk of incident hip fracture (33). In the current study, univariate analyses between the MMSE and hip fractures in the elderly cohort came out as statistically non-significant (results not shown) and was therefore not included in further analyses. Hip fracture is most often triggered by falling. The cause of falling is multifactorial in middle-aged and elderly subjects, with reduced muscle strength and poor balance among the major risk factors. These factors are also very commonly reported in COPD patients as part of the extra-pulmonary symptoms (34). Furthermore, a study in Taiwan (35) reported that hip fractures in COPD patients were more common than in controls, including risk factors such as falls, muscle weakness, and impaired balance (34). Thus, characteristics other than lung function might be of importance when estimating the risk of hip fracture in individuals with advanced airflow limitation.

Osteoporosis is recognised as one of several extra-pulmonary manifestations of COPD (5, 36, 37) and is a result of excessive bone resorption (as compared to bone formation) over time. Chronic systemic inflammation is suggested as one common cause of COPD, and osteoporosis (38, 39) is explained by a ‘spill-over’ of inflammatory mediators from local inflammation in the lungs (40). COPD-related tissue hypoxia, recurrent exacerbations, and infections are also considered as contributors to systemic inflammation (41). The inflammatory cytokines may influence the regulation of bone remodelling mainly through two different mechanisms, the osteoprotegerin (OPG)/receptor activator of nuclear factor-кB (RANK)/RANK ligand (RANKL) system and the less-understood Wnt/β-catenin signalling pathway (42). The RANKL expression on the osteoblast surface increases in response to inflammatory cytokines, thereby stimulating RANK osteoclast receptors and bone resorption. One study has suggested an association between activation of the RANKL pathway (indicating increased bone degradation) and disease activity, as well as with BMD in emphysema patients (39). In another study from Bergen, COPD patients had lower levels of OPG, a decoy receptor of RANKL and a bone protective cytokine, than controls (43).

In the current study, impaired FEV_1_ was a stronger predictor of hip fractures than the indices of airflow obstruction (i.e. FEV_1_/FVC and GOLD COPD severity definitions), which is very much in accordance with the earlier described hypothesis of chronic systemic inflammation. A low level of FEV_1_ results from a number of chronic inflammatory conditions of the lungs – not only obstructive airway disease such as COPD and chronic asthma but also restrictive disorders such as interstitial lung disease, recurrent or chronic infections, morbid obesity, severe heart failure, as well as rheumatoid diseases of the lung parenchyma and chest wall (44).

Hip fractures in elderly subjects are life-threatening events and are even more dangerous in subjects with chronic lung disease. The degree of non-reversible limitations of airflow, and in particular low levels of FEV_1_, may be useful markers of fracture risk in patients with COPD. The current study indicated that early development of non-reversible airflow limitation, before the age of 50 years, represents a particularly high risk for suffering hip fractures compared to same-age subjects with normal lung function.
